# Nicotine‐free, nontransgenic tobacco (*Nicotiana tabacum* l.) edited by CRISPR‐Cas9

**DOI:** 10.1111/pbi.13193

**Published:** 2019-07-11

**Authors:** Julia Schachtsiek, Felix Stehle

**Affiliations:** ^1^ Laboratory of Technical Biochemistry Department of Biochemical and Chemical Engineering TU Dortmund University Dortmund Germany

**Keywords:** CRISPR‐Cas9, nicotine‐free, gene editing, nontransgenic, *Nicotiana tabacum*

Worldwide, approximately 1.1 billion people are smokers and more than 7 million people die from the negative effects of smoking every year (WHO report, 2017). One of the main natural ingredients causing dependence on tobacco is nicotine. Tobacco with a lowered nicotine content could help people to overcome their nicotine addiction. Nicotine‐free (or nicotine reduced) cigarettes may contribute to reduce the number of smokers and nicotine consumption, thus reducing the risk of death from tobacco use. Most genes involved in the nicotine biosynthesis in tobacco are known and well characterized (Dewey and Xie, 2013). This opens the possibility to employ genetic engineering approaches to alter the alkaloid content of the plant, and in particular to reduce the nicotine content. Nicotine itself is composed of a pyrrolidine and a pyridine ring, which are synthesized in independent pathways (Figure 1a). Recent approaches dealt with the silencing of upper pathway genes encoding the putrescine *N*‐methyltransferase (PMT) or A622, a phosphatidylinositol‐4‐phosphate (PIP)‐family member of NADPH reductases. The applied RNA silencing methods either resulted in the increased biosynthesis of other alkaloids like anatabine (i.e. Wang *et al*., 2009) or were only successful in hairy root cultures and BY‐2 cells, but not in whole plants (Kajikawa *et al*., 2009). The final oxidation step in the biosynthesis of nicotine, as well as anatabine and anabasine, is proposed to be catalysed by flavoproteins of the berberine bridge enzyme‐like (BBL) family (Kajikawa *et al*., 2011). The knockdown of the three most highly expressed *BBL* genes (*BBLa*–*BBLc*) by RNAi or the knockout with EMS‐induced mutations resulted in a reduction of the nicotine content without increasing the content of other alkaloids (Kajikawa *et al*., 2011; Lewis *et al*., 2015). Recently, the *BBL* gene family in tobacco was expanded by the identification of *BBLd.2* and *BBLe*, leading to six known isoforms (Kajikawa *et al*., 2017). Thus, the simultaneous knockout of these *BBL* genes is a promising approach to generate a nicotine‐free tobacco plant.

**Figure 1:**
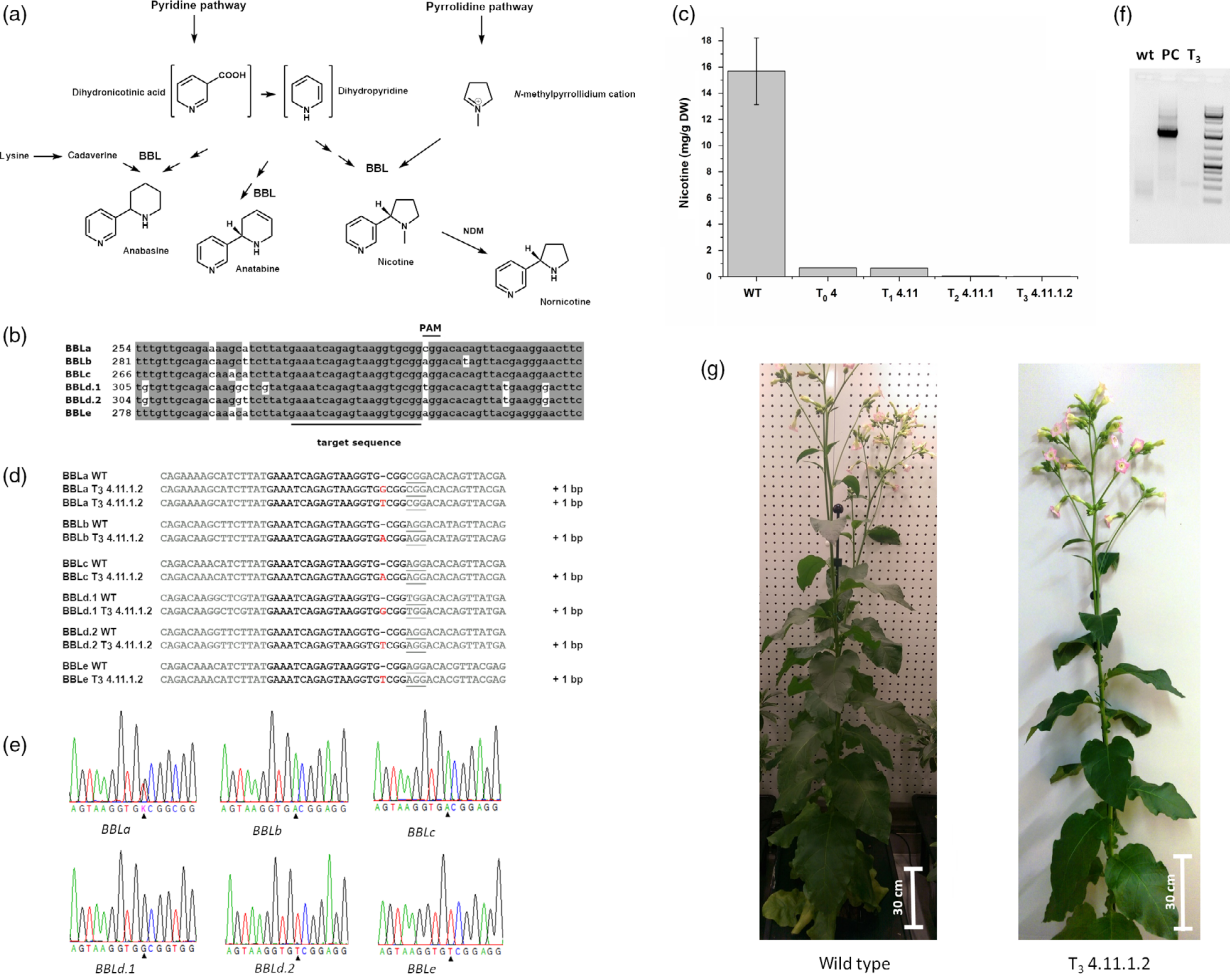
Nicotine‐free, nontransgenic *Nicotiana tabacum* l. edited by CRISPR‐Cas9. (a) Alkaloid biosynthesis pathway:Alkaloid biosynthesis consists of two independent pathways, the pyrrolidine and a pyridine pathway. Enzymes of the *BBL* gene family are proposed to be involved in the final oxidation step for the formation of tobacco alkaloids. (b) Alignment of all *BBL* gene family members: the six gene sequences of the *BBL* gene family were aligned and possible gene targets were evaluated resulting in a single target sequence identical in all six genes. (c) Comparison of nicotine content of plant 4 in all generations: The amount of nicotine was quantified with GC‐FID from 200 mg of dried and grounded leaf material of plants extracted with MTBE. Nicotine content was calculated as mg per gram dry weight (DW). (d) Genomic DNA was isolated from the plant T_3_ 4.11.1.2 and a wild‐type plant for the amplification of fragments of the six *BBL* genes; fragments were cloned into a vector for easier sequencing. One base pair insertion was observed in all six genes; for *BBLa*, two different base pair insertions could be observed. (e) Sequencing of fragments of genomic DNA of plant T_3_ 4.11.1.2 without cloning into a vector to verify the results obtained from vector sequencing. (f) Amplification of the T‐DNA cassette to test whether plant T_3_ 4.11.1.2 (T_3_) is still transgenic. As a positive control (PC), a T_0_ plant was used, as a negative control the wild type (wt). (g) Phenotype of a wild‐type plant and the nicotine‐free plant (T_3_ 4.11.1.2).

We aimed for a simple CRISPR Cas9‐based knockout strategy and searched for an identical target sequence present in all six published coding sequences (*BBLa*,* BBLc*,* BBLd.2* originated from *Nicotiana sylvestris; BBLb*,* BBLd.1*,* BBLe* from *Nicotiana tomentosiformis*; Kajikawa *et al*., 2011, 2017) to enable the use of a single‐guide RNA. Except for the PAM sequence, the chosen target sequence is identical in all six *BBL* sequences (Figure 1b). Mismatches to other sequences of the *N. tabacum* genome were excluded by BLAST search. We cloned the 20 base pair target sequence between the ubiquitin 6‐26 promoter from *Arabidopsis thaliana* and the chimeric sgRNA. The gene cassette was subsequently transferred into the transformation vector pCas9‐TPC carrying a *bar*‐gene as selection marker (Fauser *et al*., 2014).

After transformation in *Nicotiana tabacum* l. plants ‘Virginia Smoking Tobacco’ (Strictly Medicinal Seeds LLC, United States), we regenerated ten plants, denoted them as T_0_ generation and analysed the plants with regard to their nicotine content. Extraction of alkaloids was done from grounded leaves, and nicotine levels were analysed with GC‐FID. The change in nicotine levels ranged from unchanged (T_0_ 5) over a medium reduction of 65% (T_0_ 3) to a reduction of around 95% (T_0_ 1 and T_0_ 4) compared to the wild type, indicating that not all *BBL* loci were knocked out. Sequencing the fragments of genomic DNA from T_0_ 1 and T_0_ 4 plants showed no editing of *BBLe*, whereas all other *BBL* genes were mutated. The T_0_ plants 1, 3 and 4 were chosen for further characterizations in following generations. Rooted plantlets were cultivated in a plant chamber for self‐pollination in order to produce T_1_ seeds. To enable further gene editing with CRISPR Cas9, we selected transgenic T_1_ plants with phosphinothricin (PPT). Transgenic T_1_ plants were cultivated until flowering and T_2_ plants were grown from collected seeds. This growing cycle was continued to obtain T_3_ plants. While the nicotine content in plant T_1_ 1.2 did not decrease further, a decrease in nicotine content by 95% was observed for plant T_1_ 3.1. The nicotine level of plant T_1_ 4.11 was as low as in the T_0_ generation.

In order to identify nicotine‐free tobacco plants carrying knockouts in all six *BBL* genes, progenies of plant T_0_ 4 up to generation T_3_ were screened initially with regard to their nicotine content. The GC analysis of the nicotine content of the analysed T_2_ and T_3_ plants resulted in minimal peaks with retention times identical to the nicotine standard. To ensure the correct identification of the peak as nicotine, a GC‐MS measurement was performed. A m/z of 162.23 identical to nicotine was detected with a signal‐to‐noise ratio intensity of nearly 1:1. Since the signal‐to‐noise ratio of the peaks was too low for an automated peak detection, a manual analysis of the peak area was performed for estimation of the residual nicotine content. It was calculated to 0.06 mg g per DW nicotine in the T_2_ 4.11.1 and to 0.04 mg g per DW nicotine in the T_3_ 4.11.1.2 plant (Figure 1c), which means a reduction of 99.6% and 99.7%, respectively, compared to the wild type. Based on these results, the plant T_3_ 4.11.1.2 was considered as nicotine‐free.

Finally, to check whether all 12 loci were knocked out by our approach, a PCR‐based method was applied. The combination of a vector‐based cloning strategy for the amplicons followed by Sanger sequencing with the direct sequencing of the PCR product renders whole genome sequencing unnecessary. The precondition for that approach was an insertion of a single nucleotide three base pairs upstream of the PAM sequence resulting in a frameshift, that is a knockout. In contrast, in *Arabidopsis* and rice, insertions or deletions of several base pairs were observed after nonhomologous end joining (NHEJ) events (Jiang *et al*., 2013). However, for *BBLa,* sequencing results showed either the insertion of the base guanine or thymine, whereas sequencing results of the other *BBL* genes always showed the same base pair insertion (Figure 1d). To confirm these results, fragments of the *BBL* genes were amplified and sequenced directly. The previous results were confirmed by analysis of the sequencing trace of the samples (Figure 1e). Except from *BBLa*, the sequencing trace of the fragments showed a distinct signal peak for the appropriate base insertion. For *BBLa*, the sequencing trace showed a double peak for thymine and guanine, which is consistent with the results from the cloning experiments, in which both base pair insertions were detected.

To prove that the nicotine‐free plant is nontransgenic, leaf discs were cut out and transferred, after surface sterilization, to MS medium with PPT for selection. As control, a transgenic T_1_ generation plant was used. After 2 weeks, leaf discs of the T_1_ plant were still green and even started to grow, whereas the leaf discs of the nicotine‐free plant died. This result was additionally confirmed with PCR using primers that bind inside the transformation cassette thereby spanning the terminator of Cas9 and the PPT gene (Figure 1f). As a positive control, genomic DNA from a T_0_ plant was used. Neither for the wild type nor for the tested nicotine‐free plant a PCR product was obtained. Thus, the nicotine‐free plant was declared as nontransgenic.

Analysis of the GC measurements showed that the alkaloids anatabine, nornicotine and anabasine were present in wild‐type extracts, but the latter two at the detection limit. The chromatogram of the nicotine‐free plant showed again traces of anabasine and nornicotine, as well as a reduced peak area for anatabine. Additionally, ^1^H‐NMR measurements verified that no substantial changes in the primary metabolism occurred, showing that the complete knockout of the *BBL* gene family had no negative impact on other biosynthetic pathways using the tested growth conditions. Finally, no changes in the phenotype were observed (Figure 1g).

## Conclusion

The generation of a nicotine‐free and nontransgenic tobacco plant enables the introgression of this plant into other tobacco varieties and the reduction of overall nicotine and alkaloid content. Furthermore, the use of a single gRNA for the complete knockout of all *BBL*‐relevant genes makes it easy to apply this method to other tobacco varieties and species, for example *Nicotiana benthamiana* that are commonly used as heterologous production platforms. This will expand the production spectrum of *N. benthamiana* to the biotechnological production of plant‐made pharmaceuticals beyond antibodies.

## Author contributions

F.S. conceived the study. F.S and J.S designed the experiments and wrote the manuscript. J.S. performed the experiments. All authors read and approved the final manuscript.

## Conflict of interest

The authors declare no competing financial interests.
